# Acute confusional state vs psychological and behavioral symptoms of dementia. About a case

**DOI:** 10.1192/j.eurpsy.2025.1797

**Published:** 2025-08-26

**Authors:** A. Alcantara Gutierrez, L. Soldado Rodríguez, M. Martos Castello, I. Contreras Perez, A. Jurado Arévalo, E. Perdiguero Sempere

**Affiliations:** 1 Psichiatry, Hospital Universitario de Jaen, Jaen, Spain

## Abstract

**Introduction:**

Acute confusional state is usually the main diagnosis we encounter in hospitalized older adults. Its incidence increases in parallel with age. Often underdiagnosed. The main characteristics are alteration in the level of consciousness and/or attention, cognitive impairment (disorientation or reduction in memory), relatively rapid onset and fluctuations. As clinical manifestations there is usually disorganization of thought, perceptual disorders, hyper or hypoactivity, alteration of sleep rhythm and alteration of mood.

Behavioral and psychological symptoms of dementia (BPSD) describes a heterogeneous group of behavioral or mood disturbances, such as agitation, anxiety, depression, psychosis, or sleep disorders. They are universal and are present in dementia of any etiology and can be observed in any phase of the disease. They are considered multifactorial and are characterized by a recurrent or sporadic course.

68-year-old female patient who went to the hospital due to the presentation of symptoms that had been going on for 3 months (memory lapses, insomnia and irritability) that had intensified in recent days, leading to the appearance of visual hallucinations, episodes of disconnection from the environment, their language becomes impoverished and a disintegrated and incoherent speech appears with difficulty in naming.

**Objectives:**

Propose differential diagnosis between confusional syndrome and psychological and behavioral symptoms of dementia.

**Methods:**

Analytical: elevation of acute phase reactants.

Cranial CT: no findings that justify the diagnostic suspicion.

Neurology: despite the sudden onset of the symptoms, they rule out performing LP, given the unremarkable nature of the rest of the complementary tests.

It was decided to admit him to mental health to study the condition. The evolution is characterized by a fluctuating picture while the acute phase reactants begin to gradually increase, D-Dimer: 5500 and an ECG with an S1Q3T3 pattern. CT angiography is requested (ruling out PET)

Finally, after evaluation by neurology in which new analysis is requested along with urine and culture (+ for C.Glabrata) due to fever peak, lumbar puncture and cranial MRI (small acute ischemic lesions). Finally, it was decided to admit him to neurology with targeted treatment, resulting in resolution of the condition.

**Results:**

Confusional syndrome with a subacute course of etiology under study.

**Conclusions:**

BPSD of dementia are multifactorial and include different neurobiological factors, as well as environmental, social and psychological factors. They generally have a recurrent or sporadic character and can appear in all phases of any type of dementia, although they do not usually occur acutely. Therefore, it is important not to lose sight of carrying out complementary tests and a multidisciplinary approach, given that psychopathology of acute-subacute onset should not make us forget to rule out an acute confusional state.

**Disclosure of Interest:**

None Declared

